# Diffusion Tensor Imaging Tractography in Pure Neuritic Leprosy: First Experience Report and Review of the Literature

**DOI:** 10.1155/2016/2767856

**Published:** 2016-09-21

**Authors:** Michele R. Colonna, Giuseppe Tallarida, Francesco Stagno d'Alcontres, Salvatore Noto, Aurora Parodi, Alberto Tagliafico

**Affiliations:** ^1^Plastic Surgery, Department of Human Pathology, University of Messina, Messina, Italy; ^2^Plastic Surgery, University of Messina, Messina, Italy; ^3^Private Dermatology Practice, Bergamo, Italy; ^4^Department of Health Sciences, University of Genoa, Genoa, Italy; ^5^Department of Experimental Medicine, University of Genoa, Genoa, Italy

## Abstract

Five years after both right ulnar and median nerve decompression for paraesthesias and palsy, a patient, coming from Nigeria but living in Italy, came to our unit claiming to have persistent pain and combined median and ulnar palsy. Under suspicion of leprosy, skin and left sural nerve biopsy were performed. Skin tests were negative, but Schwann cells resulted as positive for acid-fast bacilli (AFB), leading to the diagnosis of Pure Neuritic Leprosy (PNL). The patient was given PB multidrug therapy and recovered from pain in two months. After nine months both High Resolution Ultrasonography (HRUS) and Magnetic Resonance Imaging (MRI) were performed, revealing thickening of the nerves. Since demyelination is common in PNL, the Authors started to use Diffusion Tensor Imaging Tractography (DTIT) to get better morphological and functional data about myelination than does the traditional imaging. DTIT proved successful in showing myelin discontinuity, reorganization, and myelination, and the Authors suggest that it can give more information about the evolution of the disease, as well as further indications for surgery (nerve decompression, nerve transfers, and babysitting for distal effector protection), and should be added to traditional imaging tools in leprosy.

## 1. Introduction

In the last few years, neuroimaging has achieved important breakthroughs in the diagnosis and care of both Central Nervous System (CNS) and Peripheral Nervous System (PNS) diseases.

In particular the role of Ultrasonography (US) and MRI has been pointed out in several diseases and in leprosy [1–5]. Nowadays, tractography has been applied to CNS extended to peripheral nerves, acting as a unique tool because of its capacity to visualize fibers and myelin anomalies [4–7].

PNL has been hypothesized and proven as a demyelinating condition [8–15].

In a patient, whose both median and ulnar nerve had been decompressed five years before, a late diagnosis of PNL was made through the classical workup: a nasal swab and slit-skin smear were negative, but a sural nerve biopsy was positive for AFB in Schwann cells (SCs) (September 2013). She was given PB multidrug therapy and recovered from pain after two months. At nine-month follow-up traditional nerve imaging through HRUS and MRI were performed.

Moreover, in this case we decided to use DTI to investigate whether myelin repair occurs after nerve trunk decompression.

DTI is an advanced, noninvasive MRI technique based on the identification,* in vivo*, of the water molecular flux tracts molecules which move in a fluid as a result of thermal energy (Brownian motion). They normally diffuse along the major axis of a longitudinal structure, such as a peripheral nerve. The presence of myelin layers directs water motion along the axis of the nerve fibers, an event which is visualized and measured by DTI using special fiber-tracking.

This technique was first applied to neural tracts in CNS, but soon it became a useful tool in peripheral nerve imaging, as it overcame T2-weighted MRI in discriminating between a nerve containing living axon fibers and that suffering from trauma or ischemia/compression with fiber damage [4–7].

In particular, variations in myelin are well documented by DTI and for this reason it has already been included in preoperative work to improve neurosurgical planning [6, 7, 9, 10], with morphological and functional images.

Compared to traditional imaging methods, DTIT appeared to be very useful detecting signs of myelination in the right median nerve of this patient.

The Authors suggest that this noninvasive technique could be further employed to correlate clinical and anatomical findings in PNL and in other leprosy forms, adding also useful data for surgery.

## 2. Materials and Methods

A 41-year-old female born in Nigeria, but living in Turin (Italy) with a normal general medical examination, had suffered for 5 years from hypoanesthesia and palsy of the right hand; neurolysis of right median nerve at the wrist in 2008; neurolysis of right ulnar nerve at the elbow in 2010.

Five years later, she was referred to our unit for persistence of pain and combined median and ulnar claw hand with the suspicion of leprosy.

A nasal swab and a slit-skin smear examination of the ear lobes, elbows, and knees were performed. A bilateral ENGraphy of the sural nerve utilizing needle electrodes and a left sural nerve biopsy were performed.

After diagnosis (September 2013), a standard PB multidrug therapy was given, and clinical and laboratory tests follow-up were performed monthly up to 12 months.

At nine months we went through nerve imaging and studied the right median and ulnar nerve proximal and distal to the elbow and the wrist.

MRI examination was performed using a clinically available 3.0 T MRI scanner. Sequences were acquired on axial planes with T1-weighted TSE and T2-weighted TSE with fat saturation.

An axial DTI sequence was then added. Diffusion was measured in 32 directions; the *b*-factor was 1,000 smm/s. The image acquisition parameters were as follows: TE = minimum, TR  = 16,675.0 ms, phase encoding R/L, frequency = 96, phase = 128,32 transverse slices were obtained without any spacing. FOV was 18 cm, slice thickness = 2 mm, gap = 0, and slice number = 49 [4].

Since then (June 2014), she has been refusing any invasive study and care we proposed; up to date (July 2016) she was called but did not come for follow-up: neither tendon transfers and microneurovascular muscle transfers for definitive care of denervated distal muscle wasting nor electrophysiological needle tests were more possible.

## 3. Results

A nasal swab and a slit-skin smear examination of the ear lobes, elbows, and knees were negative for AFB.

ENGraphy of the sural nerves showed normal amplitude. Conduction speed was at the lower limit of normal on the right and no signal was present on the left.

**Figure 1:**
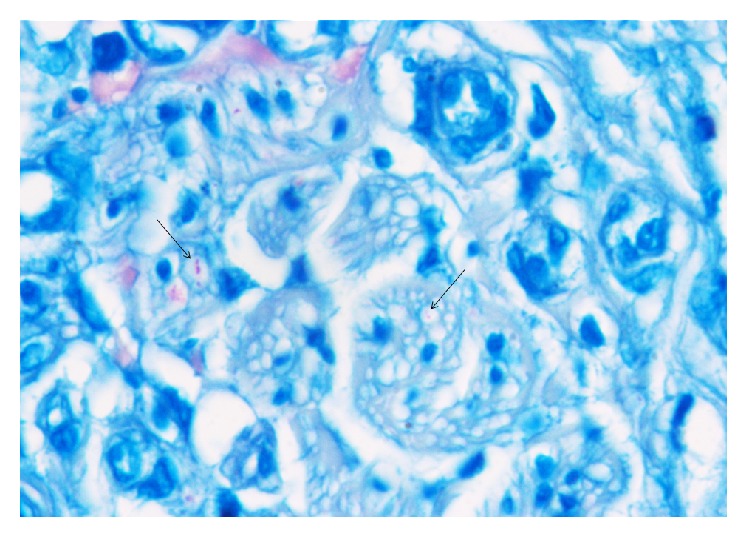
Sural nerve biopsy: Fite-Faraco stain 100x oil. Fragmented acid-fast bacilli are detectable in Schwann cells (indicated by the arrow). Courtesy of A. Clapasson.

Left sural nerve biopsy revealed AFB in SCs (Figure 1) and the final diagnosis was PNL (September 2013).

The patient started the conventional PB multidrug therapy.

HRUS (June 2014) showed the following: right median nerve thickened and was losing its fascicular structure, with a Cross Square Area (CSA) 10 smm proximally to the elbow, distally to the elbow 16 smm. Right ulnar nerve thickened and was losing its fascicular structure, with a CSA 15 smm proximally to the elbow. Epitrocleoolecranic canal was 13 smm, distally to the elbow 8 smm. Left ulnar nerve was looking almost normal. Left median nerve thickened and was losing its fascicular structure, with a CSA 16 smm proximally to the elbow, proximally to the wrist 9 smm with a bifid aspect. There was bilateral thickening of the transverse carpal ligament. Left tibialis posterior thickened and was losing its fascicular structure, with a CSA 50 smm. Both sural nerves maintained their normal structure, with a superficial lateral branch of the left sural nerve looking thickened.

Reference standards were those reported in the literature [2, 3].

MRI (June 2014) showed a marked thickening of the epiperinevrium with a spindle-like aspect of right median nerve and the largest cross-sectional area of 16 mm at the forearm was highlighted (note that the normal values of cross-sectional area should be lower than 10 mm^2^); it preserved its fascicular structure in the distal part of the forearm.

In our case study, the DTI reconstruction (June 2014) of the right median nerve trunk showed rearranging fibers, which possibly indicate the presence of myelination all over its length in the forearm. Small irregularities, typical of focal myelin deposition, just below the elbow and above the wrist were noted.

**Figure 2:**
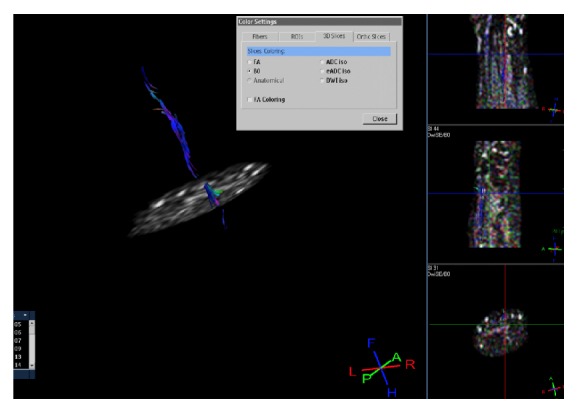
Fiber-tracking reconstruction of median nerve shows an anatomical visualization of the fibers which are oriented from proximal to distal direction, as expected (in blue). Small (nodal) areas of signal absence are detectable in the upper proximal trunk immediately below the elbow and in the tract proximal to the wrist, whilst the signal becomes smaller after bifurcation at the end of carpal tunnel.

Orientation of fibers from the proximal to the distal part of the nerve was highlighted by a blue colour (Figure 2).

Slight (nodal) signal irregularities of the proximal tracts adjacent to the carpal tunnel related to the anatomical progression of remyelination could be referred to as a sign of proximal nerve remyelination.

## 4. Discussion

PNL accounts for 4–18% of all leprosy cases and is characterized by sensory/motor deficit in the territories of nerve trunks involved; it begins as a mononeuritis and has a late progress as a mononeuritic multispect summation; because of its insidious evolution, patients often come to the clinician when anesthesia and/or palsies have just been established and their treatment by the nerve surgeon consists of nerve decompression as well as surgery for distal effectors [9, 10].

Special considerations should be made regarding procedures for distal effectors, as reported later in this section.

Some Authors [8] have studied the nerve biopsies of 11 patients with PNL in order to search for a correlation between nerve fiber status and both clinical and electroneuromyographic presentation of the disease.

SCs are the main cell population infected by* M. leprae* in the nerves and the infection triggers changes in the SCs phenotype from a myelin producing to a nonproducing state [11–13].

The nature of demyelination in leprosy has been widely debated. Some Authors have speculated that* M. leprae* has a direct effect on nerve fibers through the action of laminin 2 isoform and alpha-dystroglycan that constitute a great part of SCs basal lamina [9, 14, 15].

It has been observed that 9-O-acetyl GD3 immunoblockage,* in vitro*, reduces the* M. Leprae* adhesion [11].

Other Authors have speculated that demyelination is mediated by the stimulation of the immune system against the myelin sheath [13]

In the present study, we applied DTI for the first time, to our knowledge, to PNL.

Our results show how versatile DTI is in focusing remyelination in PNL by unifying anatomical data with clinical and neurophysiological findings. Just as in peripheral nerve surgery, its role in detecting the progression of myelination could be applied to the follow-up of decompressed nerve trunks even in PNL.

Unfortunately, after diagnosis and multidrug therapy, the patient refused any invasive studies and care and did not come for the last follow-up in July 2016; at nine months (June 2014), we decided to perform nerve imaging.

We did not apply DTIT before treatment (imaging was chosen as noninvasive follow-up) and have not got electrophysiological data but performed ENGs of both sural nerves before treatment. However, from a clinical point of view, combined ulnar and median palsy with a claw hand and complete intrinsic muscles wasting were established five years ago: damage and distal hypotrophy were yet well-established, and after PB multidrug therapy a recovery from pain was registered in two months.

We were able to study morphology of both median and ulnar nerves comparing traditional imaging and adding some interesting details on myelination in DTIT nine months after diagnosis.

An interesting debate could also result concerning small focal (“nodal”) irregularities occurring longitudinally in the nerve trunk; they might be the effect of late myelination.

That is, even if myelination of affected trunks does restart, is the focal disease present? What should be its status?

Maybe the answer could be found in the timing of surgery especially in proximal nerve trunk injuries and an early surgical babysitting of motor and sensate distal effectors along with a distal neurotization which nowadays is recommended in case of anastomosis in order to avoid distal effector atrophy [16, 17].

Thus, DTIT could also be useful in timing of nerve surgery in PNL.

However, further studies and related biopsies are needed to understand these data.

With respect to traditional MRI T2 images, it is now possible to obtain more information through DTI about fibers status and a semiquantitative study of myelinated fibers during both degeneration and regeneration.

Possibly DTI has its role in the early detection and quantitative analysis of anatomical pathological changes. It allows the calculation of the fractional anisotropy, and when it is reduced, it indicates that there is neural degeneration even without changes in conventional anatomical sequences. However, we need more studies to make the correlation of the images and pathological findings. In leprosy neuropathy, it could be important especially to differentiate chronic neuropathy (sequel) from acute lesions (neurites).

## 5. Conclusions

Because of its versatility in detecting both nerve fibers and myelin damage, we suggest that DTI could be useful in studying PNL progression from its early stages. Moreover, as it is capable of showing progression in fiber repair and remyelination, it could be also used in leprosy damage follow-up and indications for surgery. Since this is the first report of its kind, further applications should be required.

If US has been marking the modern era of the neuroimaging in leprosy and its use at high resolution is still recommended for PNL imaging [1], DTI could theoretically mark the future. We acknowledge that the costs of US compared to MRI are more favourable. However, US is not able to assess neural fibers regeneration and is an anatomical technique, whereas DTI MRI is also a functional technique.

Due to cost-related applicability in endemic areas, we suggest that tractography MRI should be present in Hub centers (important hospitals in larger towns) where even people from the surrounding areas should have access to it; these Hub centers should include all facilities required to take care of leprosy, including a complete service for peripheral nerve diseases diagnosis (electrophysiology as well as imaging) and care (clinical neurology and peripheral nerve surgery center).
